# Timely management of COPD exacerbations is associated with limited acute deterioration and early recovery: a prospective observational study

**DOI:** 10.1186/s12931-026-03802-3

**Published:** 2026-07-11

**Authors:** Rainer Gloeckl, Klaus Kenn, Daniela Kroll, Tessa Schneeberger, Inga Jarosch, Michael Wittenberg, Wolfgang Hitzl, Jing Claussen, Paul Jones, Claus F. Vogelmeier, Rembert Koczulla

**Affiliations:** 1 Institute for Pulmonary Rehabilitation Research, Schoen Klinik Berchtesgadener Land, Schoenau am Koenigssee, Germany; 2 Philipps-University Marburg, German Center for Lung Research (DZL) Universities of Giessen and Marburg Lung Center (UGMLC), Marburg, Germany; 3 Coordinating Center for Clinical Trials of the Philipps-University of Marburg, Marburg, Germany; 4 Department of Ophthalmology and Optometry, Paracelsus Medical University Salzburg, Salzburg, Austria; 5 Research Program Experimental Ophthalmology & Glaucoma Research, Paracelsus Medical University, Salzburg, Austria; 6 Medical Affairs, GlaxoSmithKline, Munich, Germany; 7 Research and Development, GlaxoSmithKline, London, UK; 8 Department of Medicine, Pulmonary and Critical Care Medicine, German Center for Lung Research (DZL), Philipps-University, Marburg, Germany; 9 Teaching Hospital, Paracelsus Medical University, Salzburg, Austria

**Keywords:** Acute exacerbation (AECOPD), Clinical course, Chronic obstructive pulmonary disease, Disease management, Early recognition, Patient-reported outcomes (PROs), Prospective study, Pulmonary rehabilitation, Recovery trajectory, Symptom burden

## Abstract

Acute exacerbations of COPD (AECOPDs) are critical events often associated with prolonged and incomplete recovery. Early recognition and prompt treatment of AECOPDs, which is rarely achieved in routine outpatient care, may influence the acute course and recovery trajectory. This study aimed to characterize the clinical course and recovery trajectory of AECOPDs detected early and treated immediately.

In this prospective, observational study, COPD patients were enrolled in a closely supervised, 4-week, inpatient pulmonary rehabilitation (PR) program. Patients who developed a pulmonologist-confirmed AECOPD during PR received same-day guideline-based therapy with systemic corticosteroids and/or antibiotics. Clinical, inflammation (C-reactive protein [CRP]), lung function (forced expiratory volume in 1 s [FEV₁]), and patient-reported outcomes (COPD Assessment Test [CAT]) were assessed at PR admission (pre-AECOPD), AECOPD day 1, day 5, and post-AECOPD (PR discharge). Functional performance outcomes, including 6-minute walk distance (6MWD) and muscle strength (quadriceps and handgrip), were also assessed at baseline and post-AECOPD.

Among 355 patients undergoing inpatient PR, 57 (16%) developed an AECOPD and were included in the analysis. At AECOPD onset (day 1), CRP increased significantly to 20.8 ± 41.8 mg/L (*p* = 0.032) and FEV₁ declined by 113 ± 220 mL (*p* < 0.001) compared to pre-AECOPD at baseline, while CAT scores showed no significant changes (*p* = 0.41). By day 5, CRP and FEV₁ were no longer significantly different from baseline. At PR discharge which occurred 13 ± 8 days after AECOPD onset, CAT scores were significantly better (23.6 ± 7.2 vs. 21.6 ± 7.8; *p* < 0.01), 6MWD increased (294 ± 105 m to 313 ± 126 m; *p* < 0.001), and quadriceps and handgrip strength improved (all *p* < 0.05) compared to pre-AECOPD baseline levels. No severe adverse events beyond the AECOPD occurred.

In this observational cohort, episodes of AECOPD detected and treated on the day of onset during inpatient PR were characterized by short-term deterioration, normalization of inflammatory and lung function parameters within days, and subsequent improvements in patient-reported outcomes and functional performance. These findings suggest that early recognition, immediate treatment, and continued adapted physical activity may be associated with better short-term recovery trajectories following an AECOPD.

**Clinical trial registration** ClinicalTrials.gov identifier: NCT04140097.

**Supplementary Information:**

The online version contains supplementary material available at 10.1186/s12931-026-03802-3.

## Background

Chronic obstructive pulmonary disease (COPD) is a leading cause of morbidity and mortality worldwide, with acute exacerbations (AECOPDs) representing pivotal events in the disease course. These episodes contribute significantly to disease progression, increased healthcare utilization, and reduced patient quality of life for patients [1]. Most patients experience one to four AECOPDs annually, accounting for an estimated 50% to 70% of total COPD-related healthcare costs [2]. Beyond their immediate clinical impact, AECOPDs frequently lead to prolonged and often incomplete recovery, with persistent impairments in symptoms, physical activity, functional capacity, and health-related quality of life lasting weeks to months [3].

Despite standard pharmacological management, many patients do not return to their pre-AECOPD clinical status. This incomplete recovery can lead to cumulative functional decline, increased risk of future exacerbations, and reduced quality of life [4, 5]. This underscores the importance of strategies that can reduce AECOPD severity and duration, and shorten recovery time [3].

Emerging evidence suggests that early recognition and prompt treatment of AECOPDs may limit the acute inflammatory cascade, reduce symptom burden, prevent deconditioning, and lower the risk of subsequent healthcare utilization [6, 7]. Notably, symptom changes within the first days of an AECOPD, such as increases in the COPD Assessment Test (CAT) score, are strongly associated with treatment response and recovery trajectories [8].

However, in a real-world outpatient setting, the ability to detect AECOPDs *at their very onset* is limited. Recent data suggest that there is a median delay of four to six days between symptom onset and treatment initiation [9, 10]. So symptom deterioration may go unnoticed or ignored for several days [7]. The setting of an inpatient pulmonary rehabilitation (PR) offers a unique environment with continuous clinical supervision, enabling prompt diagnosis and immediate treatment.

Therefore, the aim of this study was to characterize the recovery trajectory of patients with early-diagnosed and promptly treated AECOPDs from the day of onset during inpatient PR. Insights from this approach may inform future strategies for improving outpatient management of COPD exacerbations.

## Methods

### Study design and setting

This exploratory analysis was part of the *Predictors of Acute COPD Exacerbation (PACE)* study, a prospective, monocentric observational cohort trial conducted at the Schoen Klinik Berchtesgadener Land (Schoenau am Koenigssee, Germany). Consecutive COPD patients admitted for a 4-week inpatient pulmonary rehabilitation (PR) program were enrolled between February 2020 and April 2024. The full PACE protocol (including frequency of measurements and AE-specific assessments) was published earlier [11].

The primary aim of this analysis was to describe the clinical course and recovery trajectory of AECOPDs that were diagnosed early and treated immediately during inpatient PR. Sample size was determined by the size of the PACE cohort and the frequency of AECOPDs; no additional power calculations were performed for this exploratory sub-study.

### Participants and eligibility

Patients were recruited consecutively on admission to the PR program. Inclusion criteria were age ≥ 40 years and a physician-confirmed diagnosis of COPD (post-bronchodilator FEV₁/FVC < 0.70 and GOLD stage II–IV). Patients with an ongoing AECOPD at admission, a primary diagnosis of asthma or other predominant non-COPD respiratory disease, or inability to complete study procedures (e.g., cognitive impairment, language barrier) were excluded.

### Clinical definition, detection and management of AECOPD

Patients were reviewed face-to-face by a pulmonologist on two to five days per week, depending on clinical need, including physical examination and additional diagnostic testing when indicated. This routine was supplemented by continuous nursing and physiotherapist supervision. A pulmonologist was always on duty and available in case of clinical deterioration, including weekends. This structure enabled real-time recognition of changes in respiratory status, so AECOPDs were detected at or very near their onset. An AECOPD was defined clinically using the Anthonisen criteria (presence of ≥ 2 of the cardinal symptoms: increased dyspnea, increased sputum volume, increased sputum purulence) [12]. The AECOPD onset date (“day 1”) was determined as the first day on which the pulmonologist documented symptom worsening meeting the Anthonisen definition. AECOPD severity was categorized as mild (increased use of rescue medication only), moderate (requiring systemic corticosteroids and/or antibiotics), or severe (necessitating transfer to an acute hospital for advanced monitoring) [13].

Upon pulmonologist-based confirmation of an AECOPD, patients were immediately treated with AE-directed therapy, such as systemic corticosteroids or antibiotics in accordance with international guidelines [14]. The inpatient PR program was adapted to the patient’s condition with continuation when possible, or temporary modification and intensified monitoring as needed [11, 15].

For this analysis, recovery from an AECOPD was defined as an improvement in clinical outcomes above or equal to the individual’s baseline values.

### Outcomes

Baseline assessments on admission consisted of medical history, body plethysmography, blood gas analysis, blood samples (including c-reactive protein [CRP], Fibrinogen, D-Dimer, and eosinophils), cardiac assessment (echocardiography), patient-reported outcome measures (COPD Assessment Test [CAT], modified Medical Research Counsil scale [mMRC], 36 short-form survey instrument [SF-36], patient health questionnaire 9 [PHQ-9]) and functional tests (6-minute walk test [6MWT], 5-repetition and 1-minute sit-to-stand tests [STST], maximal isometric quadriceps and handgrip strength). During the four-week observation period, patients also completed daily symptom diaries (Exacerbations of COPD Tool [EXACT]). The pulmonologists were blinded to the patients´ EXACT data, which was not used to define an AECOPD. If a pulmonologist diagnosed an AECOPD, a predefined AE assessment schedule was performed as described below.

Because the AECOPD itself was the event of interest, it was not classified as an adverse event. Safety monitoring focused on adverse events occurring during and after the AECOPD period, especially those related to participation in supervised physical activity.

### Timing of assessments

Four assessment points were defined relative to PR admission and the onset of AECOPD.

- Pre-AECOPD (baseline): Assessments performed at PR admission, prior to the occurrence of an AECOPD.
- AECOPD day 1 (onset): Assessments performed on the day the AECOPD was clinically diagnosed by a pulmonologist according to Anthonisen criteria and treatment was initiated.
- AECOPD day 5 (early recovery): Assessments performed five days after AECOPD onset to capture short-term recovery dynamics.
- Post-AECOPD (PR discharge): Final assessments performed at discharge from the PR program.

Lung function, blood markers, and CAT were assessed at all four time points. Functional performance and additional patient-reported outcomes were assessed at baseline and PR discharge only.

For this analysis, recovery from an AECOPD was defined as returning to or improving beyond, the individual’s pre-AECOPD baseline values. This definition acknowledges that improvements beyond baseline may reflect the effects of ongoing PR.

### Statistical analyses

The data were checked for consistency and normality using the Shapiro-Wilks test. Repeated Measure ANOVA was performed to test for overall effects over time. Since no pre-defined endpoints were established, no adjustment for multiple testing was applied. Pairwise comparisons were performed for normal distributions using dependent t-tests; otherwise, dependent bootstrap t-tests based on 5,000 Monte Carlo simulations were used. A marginal homogeneity test was performed for discrete variables. All reported tests were two-sided and p-values < 0.05 were considered statistically significant. All statistical analyses in this report were performed using Cloud Software Group, Inc. (2023). Data Science Workbench, version 14, and Wolfram Research, Inc., Mathematica, version 13.1 (Champaign, IL, 2022).

Data are presented as mean ± standard deviation (SD), unless otherwise noted.

### Ethics and registration

The PACE study was approved by the Ethical Committee of Philipps University Marburg (Approval No. 61/19) and registered at ClinicalTrials.gov (NCT04140097). All participants gave written informed consent; the study complied with the Declaration of Helsinki.

## Results

A total of 355 COPD patients were included in the PACE study, of whom 57 (16.1%) developed a pulmonologist-confirmed AECOPD during inpatient PR (baseline characteristics are summarized in Table 1). The mean age of the cohort was 66.7 ± 7.4 years, with 42.1% being female. Almost all patients had comorbidities (98.2%), with orthopedic (82.4%) and cardiovascular (68.4%) conditions being the most prevalent. Most patients were former smokers (80.7%), with a mean of 37 ± 19 pack-years. The mean FEV1 was 34.2 ± 10.9% predicted. Patient-reported outcome measures at baseline showed an average CAT score of 23.6 ± 7.2 points and an mMRC score of 2.5 ± 1.3 points. Functional capacity as measured by the 6-minute walk distance (6MWD) was 294 ± 105 m.

**Table 1:** Baseline characteristics of 57 COPD patients that developed an AECOPD

General Characteristics	
Age, ys	66.7±7.4
Sex, female	24 (42.1%)
BMI, kg/m²	23.4±5.1
GOLD stages I / II / III / IV	0% / 8.8% / 52.6% / 38.6%
Current pneumococcal vaccination, n (%)	36 (63.2%)
Current influenza vaccination, n (%)	37 (64.9%)
Any comorbidity, n (%)	56 (98.2%)
Orthopedic, n (%)	47 (82.4%)
Cardiovascular, n (%)	39 (68.4%)
Metabolic, n (%)	30 (52.6%)
Respiratory, n (%)	13 (22.8%)
Gastroenterologic, n (%)	19 (33.3%)
Psychosomatic, n (%)	17 (29.8%)
Oncologic, n (%)	7 (12.3%)
Neurologic, n (%)	1 (1.8%)
Other, n (%)	4 (7.0%)
Smoking status, current/former/never, %	17.5% / 80.7% / 1.8%
packyears	36.8±19.2
LTOT, n (%)	39 (68.4%)
NIV, n (%)	14 (24.6%)
Fried Frailty Index, frail/pre-frail/robust	57.9% / 36.8% / 5.3%
Exacerbation History	
Lifetime AECOPDs, n	8.7±10.1
Lifetime AECOPDs with hospitalization, n	4.7±7.0
AECOPDs <12 months, n	2.2±2.2
AECOPDs with hospitalization <12 months, n	1.3±1.4
Medication	
LABA, n (%)	56 (98.2%)
LAMA, n (%)	56 (98.2%)
ICS, n (%)	45 (78.9%)
OCS, n (%)	9 (15.8%)
Lung Function & Blood Gas Analysis	
FEV1, l	0.96±0.36
FEV1%predicted	34.2±10.9
FEV1/FVC	50.0±10.7
RV, %predicted	249.3±74.0
pO2 at rest, mmHg	61.5±10.2
pCO2 at rest, mmHg	40.6±5.9
Cardiac Parameters	
Systolic blood pressure, mmHg	132±13
Diastolic blood pressure, mmHg	71±11
Ankle-Brachial Index	1.14±2.72
Pulse wave velocity, m/s	14.1±2.7
Blood Markers	
CRP, mg/l	5.2±6.4
Fibrinogen, mg/dl	384.7±100.5
D-Dimer, ng/ml	777.0±877.0
NT-proBNP, ng/l	381.2±1458.5
Eosinophils, cells/µl	390.5±239.9
Eosinophils, %	4.9±3.1
Patient-Reported Outcome Measures	
CAT, pts	23.6±7.2
SF-36 physical health, pts	28.2±9.0
SF-36 mental health, pts	41.2±14.3
mMRC, pts	2.5±1.3
PHQ-9, pts	9.1±5.9
EXACT, pts	46.8±10.9
Functional Capacity	
6MWD, m	294±105
6MWD, %predicted	45.8±15.5
5-rep STST, sec	13.2±4.9
1 Min STST, rep	17.5±6.4
Peak quadriceps force, %predicted	75.2±21.6
Peak handgrip strength, %predicted	78.5±19.3

*Abbreviations*: *AECOPD* Acute Exacerbation of Chronic Obstructive Pulmonary Disease, *BMI* Body Mass Index, *CAT* COPD Assessment Test, *CRP* C-Reactive Protein, *EXACT* Exacerbations of Chronic Pulmonary Disease Tool, *FEV1* Forced Expiratory Volume in 1 second, *FVC* Forced Vital Capacity, *GOLD* Global Initiative for Chronic Obstructive Lung Disease, *LABA* Long-Acting Beta-Agonist, *LAMA* Long-Acting Muscarinic Antagonist, *LTOT* Long-Term Oxygen Therapy, *mMRC* Modified Medical Research Council (Dyspnea Scale), *NIV* Non-Invasive Ventilation, *OCS* Oral Corticosteroids, *pCO2* Partial Pressure of Carbon Dioxide, *PHQ-9* Patient Health Questionnaire-9, *pO2* Partial Pressure of Oxygen, *NT-proBNP*  N-terminal pro-brain natriuretic peptide, *RV* Residual Volume, *SF-36* Short Form 36 Health Survey, *STST* Sit-to-Stand Test, *6MWD* 6-Minute Walk Distance

### Medical treatment of the AECOPD

On average, patients developed an AECOPD 11 ± 7 days after baseline assessment. Of these patients, 56 had a moderate AECOPD and one had a severe AECOPD (requiring transfer to an acute hospital on day three of the AECOPD). Fifty-six patients received oral corticosteroids (mean dose: 40 ± 5 mg for 5.5 ± 2.2 days) and 17 also received antibiotics. No additional adverse events beyond the AECOPD were observed during the AECOPD period.

### Changes in clinical and functional parameters during AECOPD

Table 2 shows changes in lung function, blood markers, patient-reported outcomes, and functional capacity during AECOPD from pre-AECOPD baseline to AECOPD day 1, day 5, and post-AECOPD.

**Table 2:** Longitudinal assessments in 57 patients with COPD that developed an AECOPD. Timing included baseline (11 ± 7 days pre-AECOPD), acute phase (days 1 and 5), and recovery (13 ± 8 days post-AECOPD)

	Pre AECOPD	Day 1 AECOPD	Day 5 AECOPD	Post AECOPD
Lung function & blood gas analysis				
FEV1, l	0.96 ± 0.36	**0.86 ± 0.33** ^***^	0.92 ± 0.35	0.93 ± 0.37
FEV1%predicted	34.2 ± 10.9	**30.8 ± 10.6** ^***^	33.2 ± 11.4	33.2 ± 12.2
FEV1/FVC	50.0 ± 10.7	51.2 ± 11.1	47.1 ± 10.2	47.4 ± 11.6
RV, %predicted	249 ± 74	**268 ± 77** ^*^	260 ± 80	252 ± 65
pO2 at rest, mmHg	61.5 ± 10.2	62.1 ± 11.3	62.9 ± 11.8	61.5 ± 9.1
pCO2 at rest, mmHg	40.6 ± 5.9	39.3 ± 5.8	**39.3 ± 5.6** ^*^	40.1 ± 5.3
Blood markers				
CRP, mg/l	5.2 ± 6.4	**20.8 ± 41.8** ^*^	6.9 ± 12.1	12.4 ± 23.9
Fibrinogen, mg/dl	385 ± 101	400 ± 125	**317 ± 80** ^***^	366 ± 92
D-Dimer, ng/ml	777 ± 877	755 ± 707	643 ± 454	993 ± 2465
NT-proBNP, ng/l	381 ± 1459	347 ± 526	368 ± 629	207 ± 257
Eosinophils, cells/µl	391 ± 240	**194 ± 225** ^***^	**187 ± 143** ^***^	**317 ± 206** ^***^
Eosinophils, %	4.9 ± 3.1	**2.4 ± 3.0** ^***^	**2.0 ± 1.7** ^***^	**3.3 ± 2.0** ^**^
Patient-reported outcome measures				
CAT, pts	23.6 ± 7.2	24.7 ± 7.8	**22.1 ± 7.6** ^*^	**21.6 ± 7.8** ^**^
EXACT, pts	46.8 ± 10.9	**54.8 ± 12.2** ^***^	46.4 ± 11.2	46.2 ± 11.6
SF-36 physical health, pts	28.2 ± 9.0	-	-	**30.5 ± 8.8** ^*^
SF-36 mental health, pts	41.2 ± 14.3	-	-	**47.6 ± 14.5** ^**^
PHQ-9, pts	9.1 ± 5.9	-	-	**7.2 ± 5.8** ^*^
mMRC, pts	2 [2–4]	-	-	**2 [1–3]** ^**^
Functional capacity				
6MWD, m	294 ± 105	-	-	**313 ± 126** ^***^
5-rep STST, sec	13.2 ± 4.9	-	-	**12.0 ± 5.1** ^**^
1 Min STST, rep	17.5 ± 6.4	-	-	**19.6 ± 7.4** ^*^
Peak quadriceps force, %predicted	75.2 ± 21.6	-	-	**81.0 ± 20.4** ^**^
Peak handgrip strength, %predicted	78.5 ± 19.3	-	-	**83.2 ± 18.8** ^*^

All significant differences between time points refer to comparisons against the baseline values

Boldface values indicate statistical significance

*Abbreviations*: *AECOPD* Acute Exacerbation of Chronic Obstructive Pulmonary Disease, *CAT* COPD Assessment Test, *CRP* C-Reactive Protein, *EXACT* Exacerbations of Chronic Pulmonary Disease Tool, *FEV*_1_ Forced Expiratory Volume in 1 second, *FVC* Forced Vital Capacity, *mMRC* Modified Medical Research Council (Dyspnea Scale), *pCO*_2_ Partial Pressure of Carbon Dioxide, *PHQ-9* Patient Health Questionnaire-9, *pO*_2_ Partial Pressure of Oxygen, *NT-proBNP* N-terminal pro-brain natriuretic peptide, *RV* Residual Volume, *SF-36* Short Form 36 Health Survey, *STST* Sit-to-Stand Test, *6MWD* 6-Minute Walk Distance

^*^p < 0.05, ^**^p < 0.01, ^***^p < 0.001; data presented as mean ± SD or median [IQR]

On AECOPD day 1 compared to baseline, FEV_1_ declined significantly by a mean of 113 ± 220 ml, C-reactive protein levels rose significantly to 20.8 ± 41.8 mg/L, and health status worsened only modestly (CAT score change of + 0.7 ± 5.9, *p* = 0.41). Patient-reported symptom burden captured by the EXACT diary showed a mean increase of 8.4 ± 9.7 (*p* < 0.001), indicating a significant but moderate worsening of daily symptoms.

**Fig. 1:**
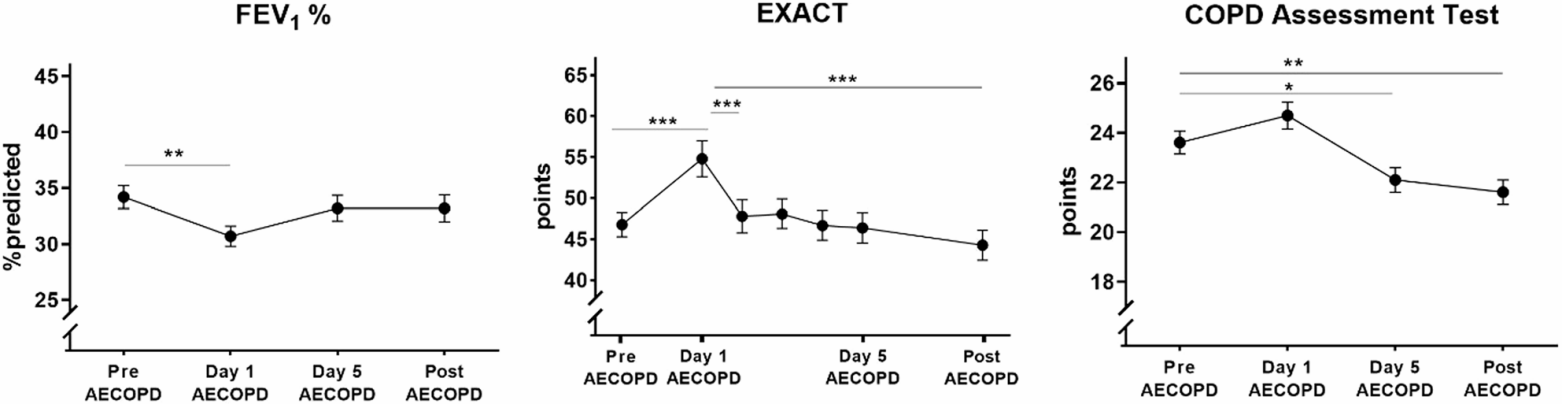
Longitudinal changes in lung function and symptom scores during timely treated AECOPD. Data represent mean (± 95% CI) for forced expiratory volume in 1 s (FEV_1_), *Exacerbations of COPD Tool* (EXACT), and *COPD Assessment Test* (CAT) in 57 patients. Assessments were conducted at four time points: baseline (11 ± 7 days pre-AECOPD), during AECOPD (days 1 and 5), and at recovery (13 ± 8 days post-AECOPD)

By AECOPD day 5, FEV_1_ showed some mean improvement, but with a wide scatter (60 ± 200 ml) and was not significantly different from baseline values (*p* = 0.067) (Fig. 1). CRP levels decreased to 6.9 ± 12.1 mg/l. Health status was significantly better than baseline (CAT score 22.1 ± 7.6 points, *p* < 0.05), and EXACT scores had returned to baseline levels.

**Fig. 2:**
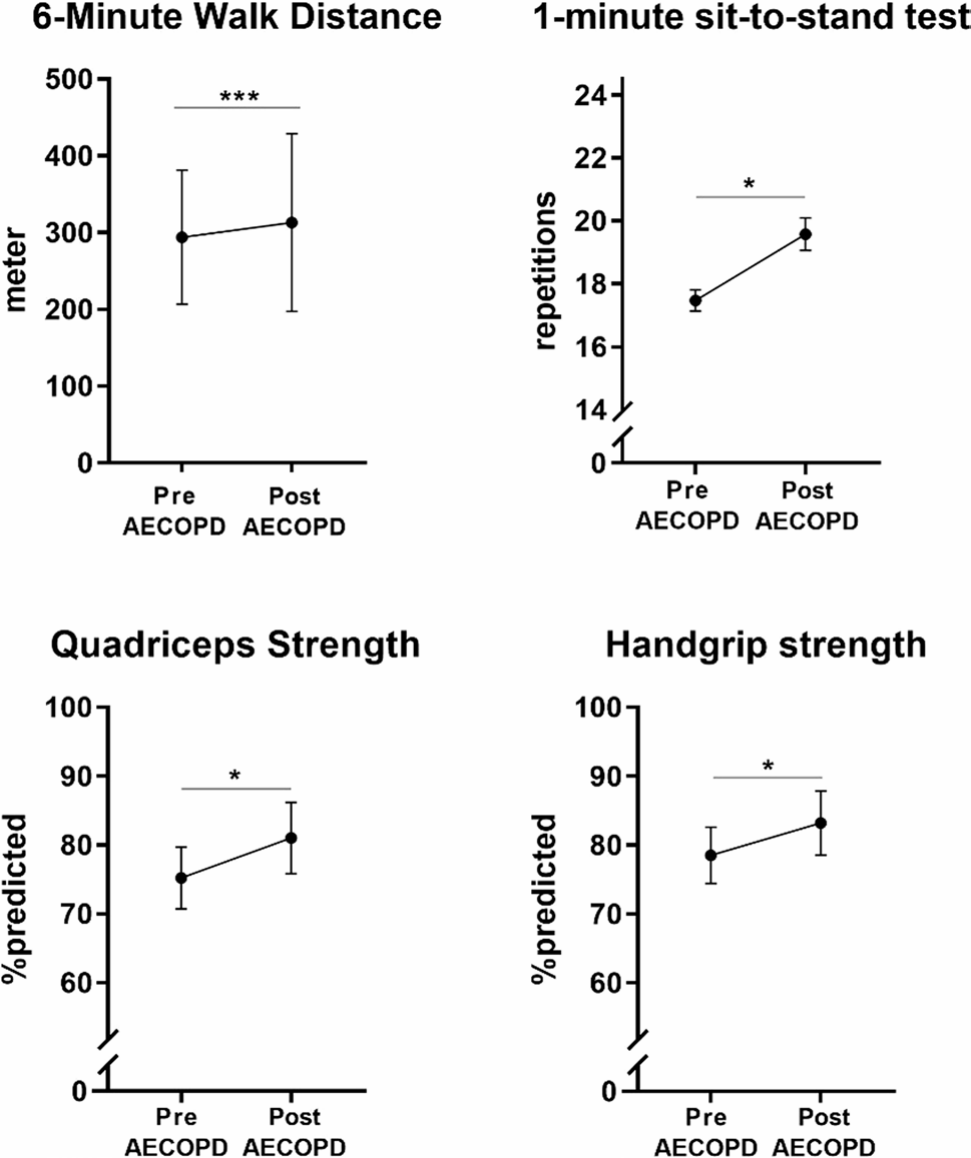
Longitudinal changes in functional outcomes relative to AECOPD onset. Data represent mean (± 95% CI) in 57 COPD patients. Assessments were conducted at baseline (11 ± 7 days pre-AECOPD) and at recovery (13 ± 8 days post-AECOPD) following a timely treated AECOPD

The second follow-up assessment (at PR discharge) was conducted on average of 13 ± 8 days after the onset of AECOPD. At that time, all outcomes showed a significant improvement or a return to the pre-AECOPD baseline (see Table 2 and Fig. 2). For contextual reference, supplementary Table S1 provides the outcomes of the 298 PACE patients who did not develop an AECOPD during the same inpatient PR program under identical conditions.

## Discussion

This exploratory, single-center study, conducted within a highly supervised inpatient PR setting, provides unique insights into the potential recovery trajectory when AECOPDs are recognized and treated immediately. Prompt recognition and same-day initiation of AECOPD-directed therapy were associated with a recovery trajectory characterized by stability within the first 5 days after onset and subsequent improvements in clinical, functional, and patient-reported outcomes. The crucial role of early recognition is supported by the literature, as a large proportion of AECOPDs go unreported yet are linked to negative long-term outcomes and increased risk of future events [7, 16]. The sooner treatment is initiated after diagnosis or symptom onset, the faster symptoms resolve, quality of life improves, and hospitalization risk diminishes, while delays correlate with poorer health-related quality of life and higher hospitalization rates and costs [17]. Further evidence shows that the risk of a cardiovascular event (i.e. acute coronary syndrome or acute cerebrovascular events) is markedly increased especially during the first two weeks after an AECOPD [18, 19]. In our cohort, no cardiovascular events occurred during the post-AECOPD observation period. Although the underlying reasons cannot be determined, the absence of significant changes in NT-proBNP levels may indicate a lower degree of acute cardiac stress in this cohort. However, this observation is exploratory and should be interpreted with caution.

Our study, performed within an inpatient PR program with close clinical supervision and daily medical contact, facilitated the detection of AECOPDs at their very onset. The ability to detect AECOPDs at or near their onset and initiate immediate therapy is in substantial contrast to typical outpatient settings, where diagnostic delays are common [3]. This timely management strategy appears to have reduced the acute inflammatory cascade and symptomatic deterioration. This is evident in the relatively modest initial symptomatic worsening in our cohort. The mean CAT score increased by only 1.1 points from pre-AECOPD to AECOPD day 1. This is in contrast to the substantial 4- to 5-point increase reported for outpatients and the 6- to 10-point increase reported for hospitalized patients [20]. In addition to CAT, daily symptom monitoring with the EXACT diary revealed a mean increase of > 8 points at AECOPD onset, indicating a significant worsening of respiratory symptoms. This rise exceeded typical minimal important differences for daily symptoms [21]. However, the subsequent fast decline in EXACT score suggests that early recognition and immediate treatment may have prevented the full escalation of symptoms normally observed in untreated cases. The divergent behavior of CAT and EXACT likely reflects differences in construct and sensitivity, with EXACT capturing acute symptom fluctuations more sensitively, while CAT reflects broader health status.

The reduced symptomatic response, alongside the prompt stabilization of FEV_1_ and CRP levels, suggests that acute deterioration may be limited by a proactive approach. Further, FEV_1_ did not change significantly in the 298 non-exacerbating PACE patients over the full PR program. This finding is consistent with well-established evidence showing that PR improves exercise capacity and quality of life in COPD patients without altering spirometric values [22, 23]. Since no FEV_1_ gain was achieved with PR, the rapid recovery of FEV_1_ to pre-exacerbation baseline within 5 days in the AECOPD group shows equivalent outcomes to patients with uncomplicated PR.

Furthermore, the follow-up assessment on PR discharge (13 ± 8 days after AECOPD onset) revealed a significant improvement in health status compared to pre-AECOPD baseline, meeting the threshold of 2-points for the minimal important difference in the CAT score [24]. These findings align with Macleod et al.‘s framework, which states that effective interventions can occur at three key time points: acutely, to attenuate the length and severity of an established exacerbation; in the aftermath, to prevent early recurrence and readmission, which are common; and in the long-term, establishing preventive measures that reduce the risk of future events [5]. Our study particularly addressed the acute phase and showed that the treatment is effective at this point.

In addition to immediate pharmacological management, the continuation of adapted physical activities under close clinical and physiotherapist supervision in the PR setting likely served as a critical co-intervention. Early PR is recognized as a safe and vital intervention that enhances recovery and functional outcomes following AECOPD [15, 25, 26]. The significant improvements observed in functional capacity (e.g., 6MWD increased from 294 ± 105 m to 313 ± 126 m) and patient-reported outcomes at PR discharge support that this controlled environment optimized delivery and adherence to physical activity during the critical recovery period, thereby reinforcing and accelerating the benefits of early treatment.

### Limitations

The primary limitation is the observational, single-arm design. Although the observed rapid recovery trajectory is favorable, the absence of a randomized delayed-treatment or usual-care control group precludes drawing causal conclusions. The observed effects are therefore an association influenced by the unique, highly optimized environment of inpatient PR. This environment enabled both immediate pharmacological therapy and continued supervised physical activity, an ideal scenario not typical of routine care. However, adaptations to the PR program during AECOPD were guided by clinical judgment and not systematically documented in a standardized format at the individual patient level, which limits the precision with which the contribution of continued adapted exercise to recovery outcomes can be quantified. Future studies should prospectively document PR modifications during AECOPD episodes to enable a more precise analysis of this relationship.

All confirmed AECOPDs in this cohort were classified as moderate or severe. Truly mild exacerbations resolving spontaneously or with rescue bronchodilator use alone before Anthonisen criteria were formally met would not have triggered the predefined diagnostic workup and may therefore be underrepresented in this analysis.

Nevertheless, this study’s strengths lie in its prospective design, well-characterized patient population, and comprehensive collection of clinical, functional, and patient-reported outcome measures.

### Future directions

Future research should explore and integrate novel strategies for the early detection and management of AECOPD into routine clinical care. Notably, the PACE study also investigated the COPD Exacerbation Recognition Tool (CERT) [27, 28]. This five-item symptom checklist was developed as an educational tool to help patients recognize symptomatic deterioration and know when to seek medical advice. Within the PACE cohort, CERT enabled patients to detect worsening symptoms one day before a clinician-confirmed AECOPD diagnosis [29]. Developing and implementing patient-centric tools like CERT can empower patients to recognize when to seek medical attention for worsening symptoms. This can promote earlier intervention, which may include prescription of `rescue packs´ (corticosteroids and/or antibiotics) for selected frequent exacerbator patients (a practice already implemented in several countries). This is particular important in parts of the world, where patients struggle to quickly see their physicians [30]. Future research should investigate the effectiveness of integrating these tools within community-based care and evaluate their impact on AECOPD outcomes.

## Conclusions

In this prospective cohort study of patients with COPD undergoing inpatient PR, clinician-diagnosed AECOPDs that received same-day AECOPD-directed therapy was associated with rapid improvements in lung function, inflammation, and patient-reported outcomes. The results suggest that early recognition and immediate initiation of therapy (in the context of a supervised inpatient PR program) may help limit acute deterioration and support recovery. Further controlled studies are necessary to determine whether the observed associations reflect the causal benefits of earlier treatment and continuing adapted, supervised physical activity during AECOPD.

## Supplementary Information

Supplementary Material 1.

## Data Availability

The datasets used and/or analyzed during the current study are available from the corresponding author on reasonable request.

## Abbreviations

6MWD: 6-Minute Walk Distance
6MWT: 6-Minute Walk Test
AE: Acute Exacerbation
AECOPD: Acute Exacerbation of Chronic Obstructive Pulmonary Disease
BMI: Body Mass Index
CAT: COPD Assessment Test
CERT: COPD Exacerbation Recognition Tool
COPD: Chronic Obstructive Pulmonary Disease
CRP: C-Reactive Protein
EXACT: Exacerbations of Chronic Pulmonary Disease Tool
FEV: Forced Expiratory Volume in 1 second
FVC: Forced Vital Capacity
GOLD: Global Initiative for Chronic Obstructive Lung Disease
ICS: Inhaled Corticosteroids
LABA: Long-Acting Beta-Agonist
LAMA: Long-Acting Muscarinic Antagonist
LTOT: Long-Term Oxygen Therapy
mMRC: Modified Medical Research Council
NIV: Non-Invasive Ventilation
NT-proBNP: N-terminal pro-brain natriuretic peptide
OCS: Oral Corticosteroids
PACE: Predictors of Acute COPD Exacerbation
pCO_2_: Partial Pressure of Carbon Dioxide
PHQ-9: Patient Health Questionnaire-9
pO_2_: Partial Pressure of Oxygen
PR: Pulmonary Rehabilitation
PROs: Patient-Reported Outcomes
RV: Residual Volume
SF-36: Short Form 36 Health Survey
STST: Sit-to-Stand Test
