# Supplementary material for: Timely management of COPD exacerbations is associated with limited acute deterioration and early recovery: a prospective observational study
Source: Respir Res. 2026 Jul 11;27:279. doi: 10.1186/s12931-026-03802-3 (PMC13355348; doi:10.1186/s12931-026-03802-3)
Supplement: Supplementary file 1 — Supplementary Material 1. [file 12931_2026_3802_MOESM1_ESM.pdf]

ONLINE SUPPLEMENT

Timely Management of COPD Exacerbations Is Associated with Limited Acute Deterioration and Early Recovery: A Prospective Observational Study

Authors:

Rainer Gloeckl, Klaus Kenn, Daniela Kroll, Tessa Schneeberger, Inga Jarosch, Michael Wittenberg, Wolfgang Hitzl, Jing Claussen, Paul Jones, Claus F. Vogelmeier, Rembert Koczulla

Table S1: Assessments of 298 COPD patients from the PACE study cohort who did not develop an AECOPD during inpatient pulmonary rehabilitation (PR).

	PR admission	PR discharge	delta	p
FEV1, l	1.18±0.52	1.19±0.52	0.01±0.17	0.26
FEV1 %predicted	40.1±14.6	40.6±14.8	0.5±5.6	0.14
FEV1/FVC	52.1±11.6	52.8±10.8	0.7±9.1	0.24
RV, %predicted	226.8±69.9	233.2±80.6	7.4±55.8	0.03
pO2 at rest, mmHg	63.8±9.0	63.9±10.3	0.1±8.9	0.89
pCO2 at rest, mmHg	38.7±5.8	39.4±6.5	0.7±5.0	0.03
CRP, mg/l	7.0±10.9	6.4±9.7	-0.6±11.2	0.35
Eosinophils, cells/µl	257±158	253±135	-4±116	0.53
Eosinophils, %	3.4±2.0	3.4±1.9	0.0±1.3	0.81
CAT, pts	20.3±7.4	18.2±7.5	-2.1±4.4	<0.001
EXACT, pts	40.7±9.0	38.4±11.2	-2.3±7.2	<0.001
SF-36 physical health, pts	30.7±9.3	33.9±14.0	3.1±11.9	<0.001
SF-36 mental health, pts	44.4±12.1	48.3±11.7	3.9±10.9	<0.001
PHQ-9, pts	8.2±5.1	6.0±4.8	-2.2±3.6	<0.001
mMRC, pts	2 [1-3]	2 [1-2]	0 [-1-0]	<0.001
6MWD, m	320±121	356±123	36±46	<0.001
5-rep STST, sec	13.1±5.8	11.0±4.3	-2.1±3.4	<0.001
1 Min STST, rep	18.8±7.4	21.6±9.0	2.8±4.7	<0.001
Peak quadriceps force, %predicted	79.7±25.8	86.5±26.8	6.8±10.6	<0.001
Peak handgrip strength, %predicted	83.4±20.6	86.8±21.1	3.4±9.6	<0.001

Data presented as mean± SD or median [IQR]

Abbreviations: AECOPD: Acute Exacerbation of Chronic Obstructive Pulmonary Disease, CAT: COPD Assessment Test, CRP: C-Reactive Protein, EXACT: Exacerbations of Chronic Pulmonary Disease Tool, FEV_1: Forced Expiratory Volume in 1 second, FVC: Forced Vital Capacity, mMRC: Modified Medical Research Council (Dyspnea Scale), pCO_2: Partial Pressure of Carbon Dioxide, PHQ-9: Patient Health Questionnaire-9, pO_2: Partial Pressure of Oxygen, RV: Residual Volume, SF-36: Short Form 36 Health Survey, STST: Sit-to-Stand Test, 6MWD: 6-Minute Walk Distance
